# Insignia of the Royal Army Dental Corps and Commonwealth Dental Corps

**DOI:** 10.1038/s41415-022-3995-2

**Published:** 2022-03-11

**Authors:** David R. Radford

**Affiliations:** Principal Lecturer, University of Portsmouth Dental Academy, University of Portsmouth, Hampshire Terrace, Portsmouth, PO1 2QG, UK

## Abstract

The insignia worn by the British and Commonwealth Armed Services are rich in symbolism and meaning to the corps and regiments that wear them. Originally, before the adoption of national uniforms pre-1700s, there was little to distinguish friend from foe. To overcome that problem, certainly in battle, it was common practice to wear some sort of distinctive emblem, such as a sprig of a native plant. This then developed, in the reign of Charles II, to the custom of individual regiments or corps adopting devices and designs of their 'colours', through to modern cap and collar badges.

On the centenary of the Royal Army Dental Corps, this paper gives some insight into those designs adopted by the Royal and Commonwealth Dental Corps, when a distinct service of dental care was recognised. They give a fascinating insight into the adoption and amalgamation of both national and dental symbols. The Dental Corps, separate from Army Medical Corps, went on to develop the vital provision of dental healthcare, both in the field and at home.

## Key points

- Illustrates the insignia worn by the Royal Army Dental Corps since 1921.
- Recognises that certain Commonwealth Armies had pre-dated the inception date of 1921 in their establishment of separate Dental Corps.
- Highlights the technical expertise of those who manufacture the cap, collar badges and sweetheart brooches.

## Introduction

Inspired by the *British Dental Journal'*s edition focusing on the Royal Army Dental Corps and its work and history during the centenary year,^1^ I thought it might be of interest to write a short article on the insignia of the corps with some additional notes on Commonwealth Dental Corps insignia. My father inherited a small collection of military cap badges from his father, who had served in World War 1. At that time and until the 1980s, collecting cap and collar badges was a common pastime, with some quite magnificent badges to be found in flea markets across the country, sometimes at the cost of only a few pounds. My father and I used to spend many a happy hour on a Saturday afternoon in Brighton's North Laines going through boxes of buttons and cards of cap and collar badges. This paper is not intended as a collector's guide but to give an insight into the variety of designs adopted and the skill of metal working craftsman who produced the cap and collar badges. When I inherited the collection of predominantly collar badges from my father at the turn of the century, I judiciously added to it to ensure that it is a 'living collection', while being mindful that the cost of badges has increased many-fold. I continue to cultivate a small specialist collection of UK and Commonwealth Dental Corps insignia, some of which are illustrated and described in this paper.

## The Army Dental Corps and Royal Army Dental Corps 1921 to date

**Fig. 1:**
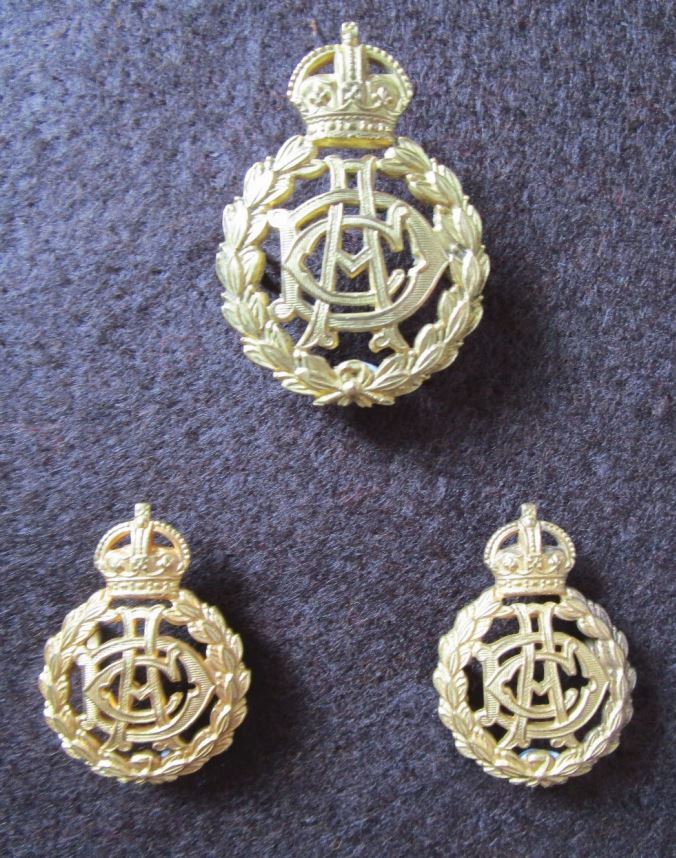
AD Corps officer's cap and collar badges, cast gilt examples (1921-1946). The intertwined letters ADC in a gothic style font surrounded by a laurel leaf wreath, surmounted with a king's crown. The king's crown (1901-1952, the Tudor crown) was used post the reign of Queen Victoria, before the use of the present queen's crown in 1952 (crown of Saint Edward) in the current reign of Queen Elizabeth II

**Fig. 2:**
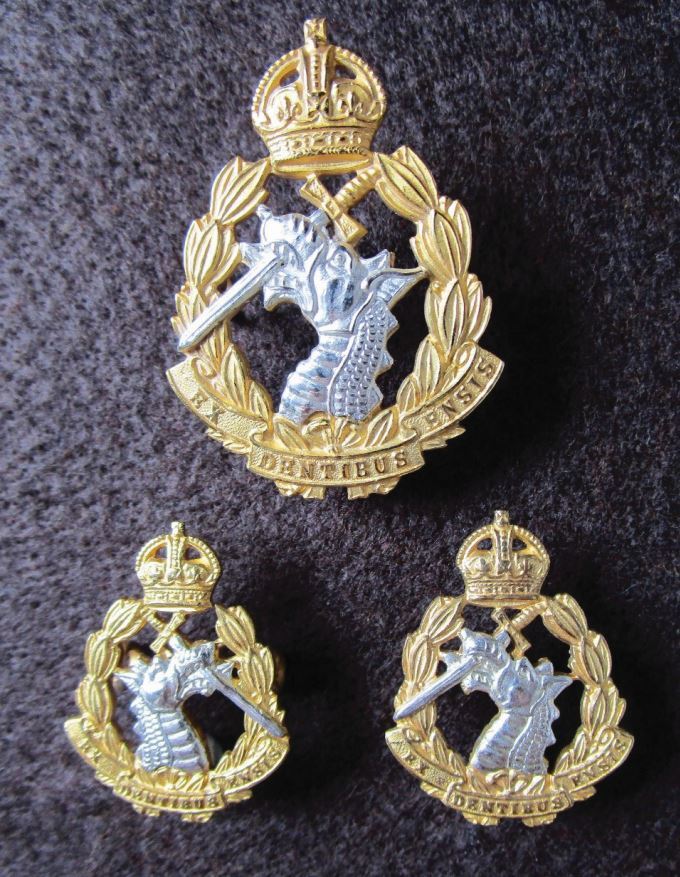
Officer's cap and collar badges in two-part silver and gilt metals (RADC 1946-1952). The in-facing dragons on the collar badges reflect the craftsmanship of the skilled technicians of the manufacturer, J. R. Gaunt of London

**Fig. 3:**
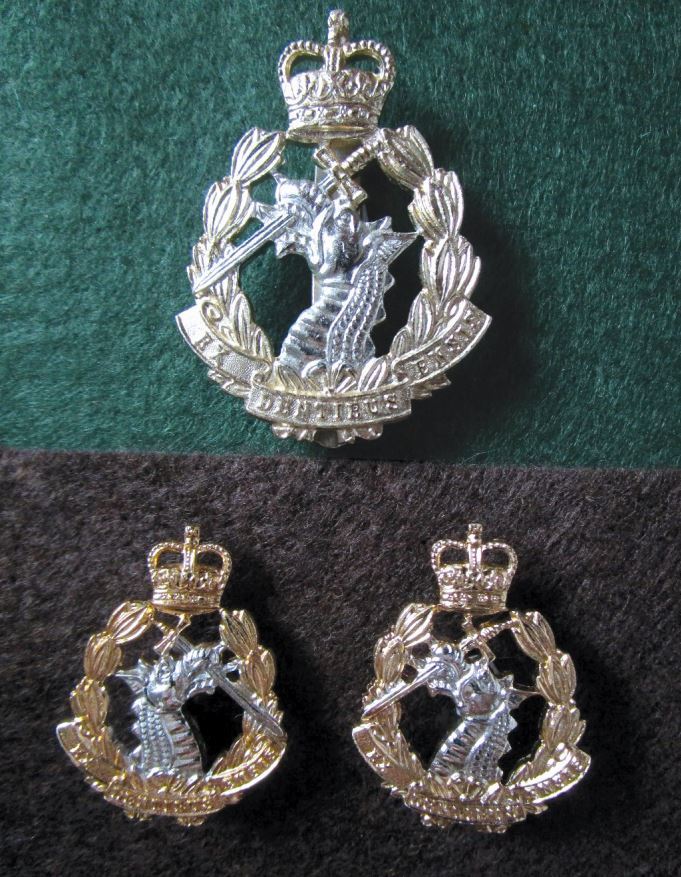
Anodised aluminium (staybright) cap and collar badges of the RADC. The cap badge is shown on a green background. Officers' and soldiers' caps have a broad green band above the peak (as seen in Figure 3 of 'The Royal Army Dental Corps today'^8^). Anodised aluminium was introduced into general service wear in the early 1950s

The British Army Dental Corps (AD Corps) was established in 1921^2^ to maintain the fighting power of the frontline troops by reducing wastage caused by debilitating dental diseases. In those days, corps' badges often simply consisted of interwoven initials of the corps, such as AD Corps, until 1946 (Fig. 1). This was similar, for example, to the Army Veterinary Corp (1903-1918) or the Army Pay Corps (1902-1920). However, in 1946, King George VI granted a Royal prerogative to the AD Corps, thus becoming the Royal Army Dental Corps (RADC). It adopted a much more imaginative design for its cap and collar badges, perhaps one of the British Army's most interesting and distinctive (Fig. 2). The design depicts the legend of Cadmus. According to Greek mythology, Cadmus found a dragon had killed his soldiers so he slayed the dragon with one blow of his sword. He heard a voice instructing him to extract the dragon's teeth and sow them in the ground. Cadmus immediately obeyed. As soon as the teeth were planted, a crop of men sprang from the soil, fully grown and armed. It is from this legend that the RADC takes its motto, 'Ex Dentibus Ensis' (from the teeth, a sword).^3^ In Figure 2, both the cap badge and a pair of officers' facing collar badges are shown. The collar badges are mirror facing, as one could not have one dragon facing backwards (in retreat). For the prosthodontists, just think of the technical expertise and skill required to produce mirror facing dies. With officers' badges finished in silver plate and gilt, they verge on true items of bespoke jewellery. The badge is still in current use with a queen's crown (Fig. 3).

## Dental Hygienist qualification badge

**Fig. 4:**
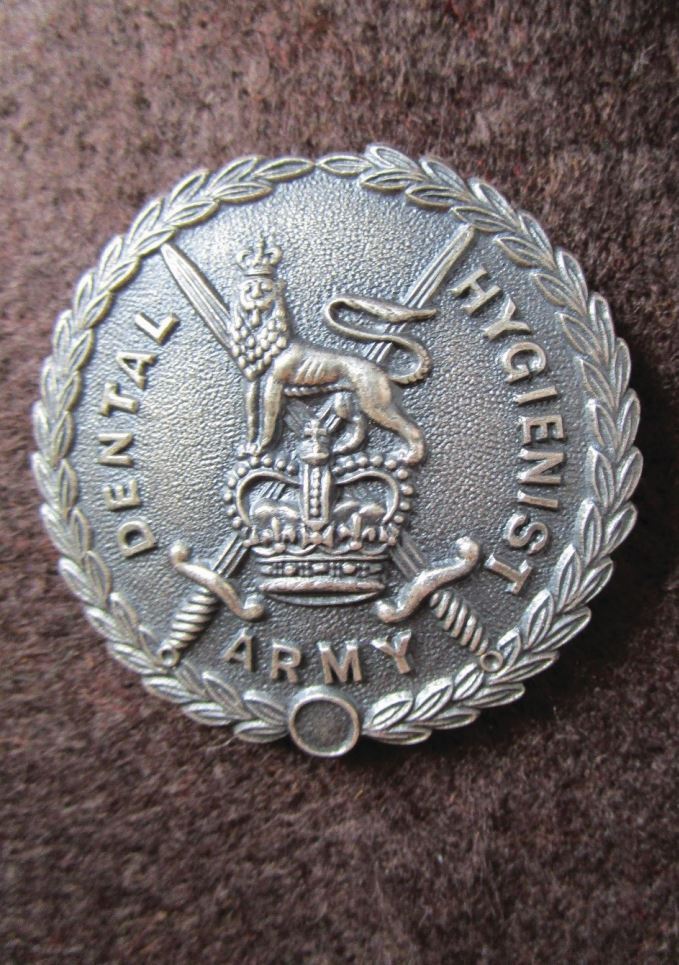
Dental Hygienist RADC qualification badge (circa 1970)

Military-trained dental hygienists also received a badge on qualification (Fig. 4). While lacking the intricacy of previous examples, it shows crossed swords and a queen's crown surmounted by a lion, the insignia of the British Army. The Tri-Service Dental Hygienist Training School closed in 2013.

## Sweetheart brooches

**Fig. 5:**
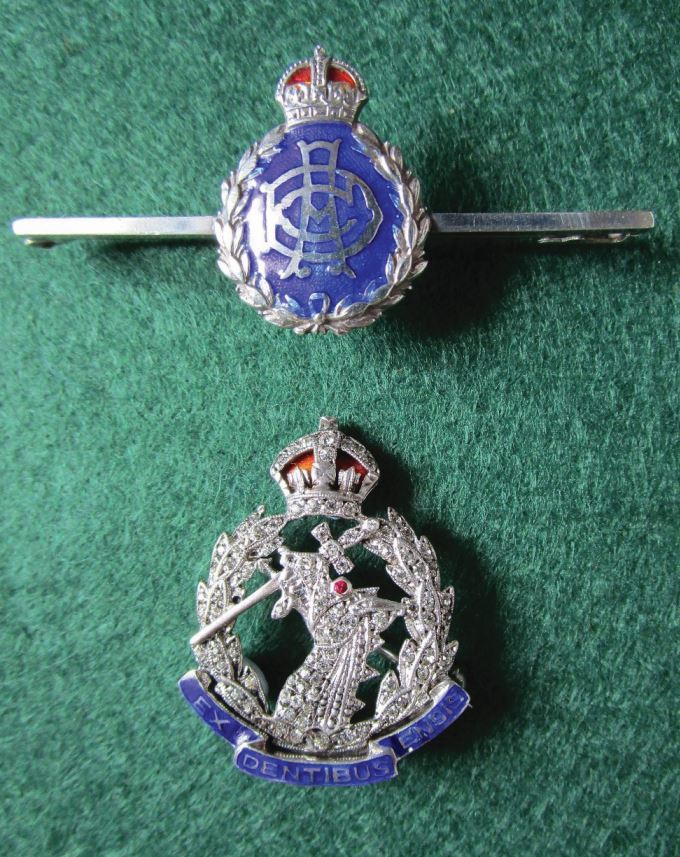
Two examples of sweetheart brooches. The top image, a less expensive example of a sterling silver bar brooch of the AD Corps (circa 1939). The lower image is of a more expensive silver and enamel RADC with paste diamonds - note the ruby eye of the dragon (1948-1952)

Sweetheart brooches have been in existence for over 150 years, with design fashions ebbing and flowing.^4^ These ornate, miniaturised copies of regimental badges were bought by husbands and sons, whatever their rank and were given to mothers, wives and girlfriends; thus, the name 'sweetheart' brooches. The cost of these to the individual varied widely due the materials used (such as 18 carat or 9 carat gold, silver, base metal, tortoise shell, mother of pearl, enamels, paste diamonds and real diamonds) and the quality of finish and size of production. For example, in 1939, good-quality silver and enamel brooches were retailed at 6 shillings and 6 pence (33 pence), solid 9 carat gold at £2 and 10 shillings (£2.50) and 18 carat gold and platinum, inset with rose diamonds, in the region of £25. Those badges purchased now by collectors, dependant on their rarity and the interest in the particular regiment or corps, would be between £15-30 or £60-90, respectively and if one could find a diamond and platinum example in pristine condition, it could have a value of thousands of pounds (Fig. 5).

## Commonwealth insignia

### Canadian Army Dental Corps

**Fig. 6:**
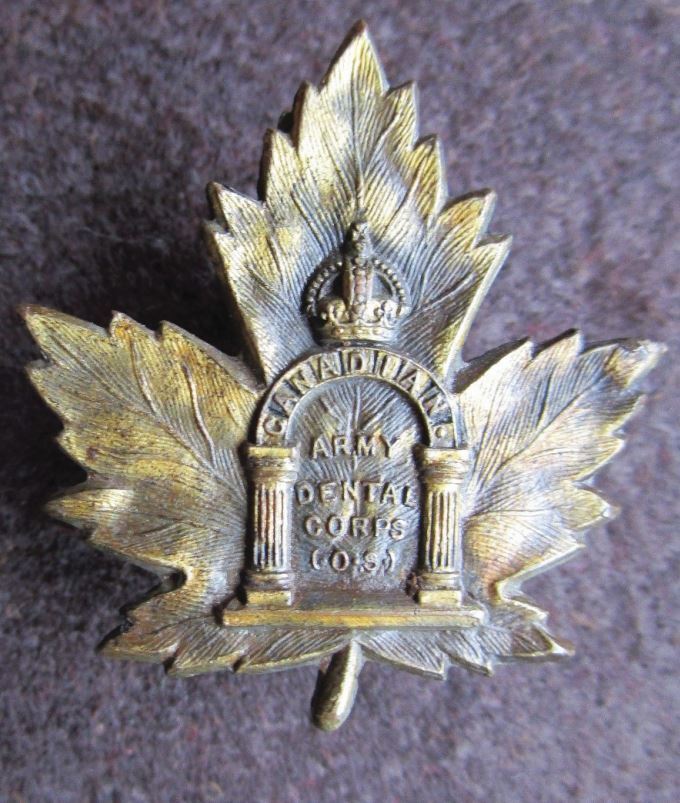
Officer's bronze cap badge of the Canadian Army Dental Corps (1915-1928). The Canadian maple leaf was a common design of the Canadian Expeditionary Forces during and after the First World War. The arch depicted in the centre of the badge was taken from the Sublime Degree of the Holy Royal Arch Masons and is symbolic of the mouth, the 'chief entrance' to the human body

The Canadian Army Dental Corps was established in 1915 and received a Royal designation granted by Royal prerogative in 1947.^5^ The striking maple leaf design adopted by the Corps at the time of the First World War is illustrated in Figure 6. The ensuing history of the Corps is complex; however, this is recorded in the 'History and heritage of the Royal Canadian Dental Corps', published in its centenary year 2015^5^ and the comprehensive detail is outside the scope of this paper.

### Australian Dental Corps

**Fig. 7:**
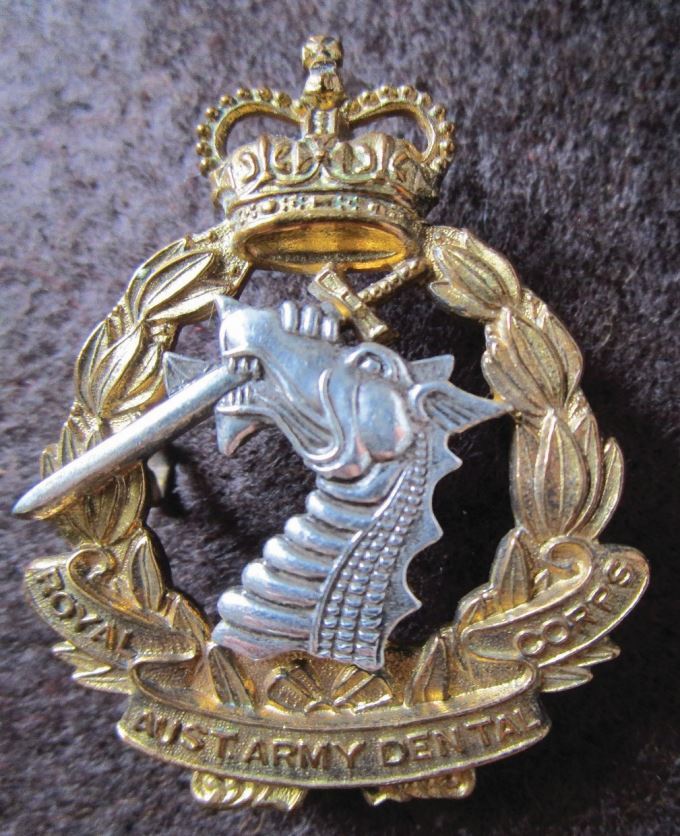
Royal Australian Dental Corps, silver gilt officer's cap badge with a queen's crown (post-1952), adopting a similar design of the British RADC but the scroll superimposed on the wreath is 'Royal Aust. Army Dental Corps' rather than a motto

The Australian Dental Corps was established in 1943 and received a Royal designation in 1948. There is a detailed and well-referenced account of dentistry online on the Australian Armed Services of the twentieth century^6^ (Fig. 7).

### New Zealand Dental Corps

**Fig. 8:**
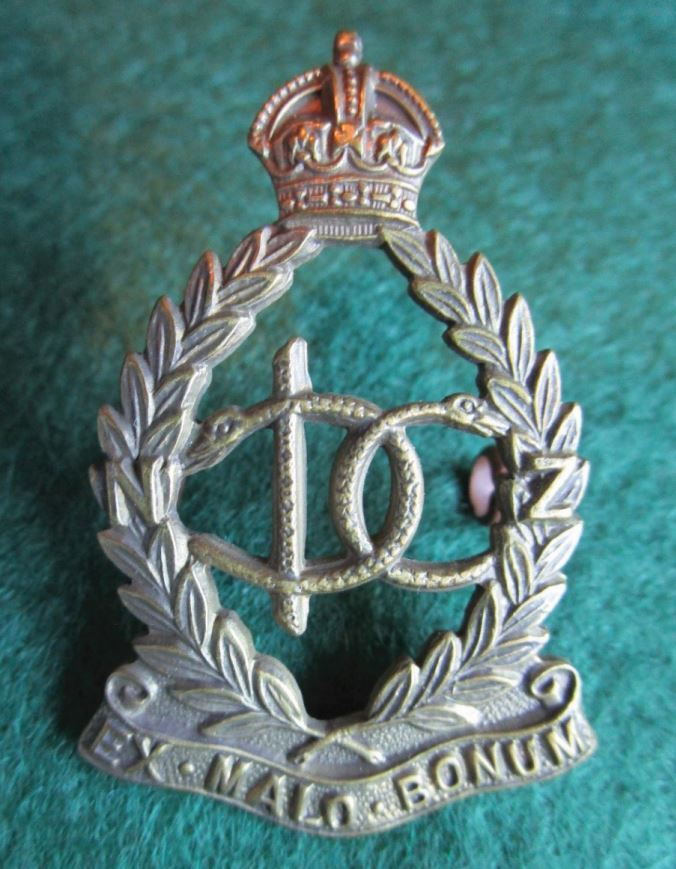
The New Zealand Dental Corps 1915-1947. Note the two serpents with the staff making the initials of 'DC' but also the technical expertise of the voided centre of the badge

Similar to the Canadian Army Dental Corps, the New Zealand Dental Corps was raised in 1915 and was granted a Royal designation in 1947. The insignia adopted in 1915 is an unassuming badge but when studied in greater detail, a fascinating one (Fig. 8). In the centre of the laurel wreath are two intertwined serpents, with a staff (the rod of Asclepius) placed to one side, thus spelling 'DC' for 'Dental Corps'. The letters N and Z are super-imposed on the laurel wreath at the 9 and 3 o'clock positions. The motto 'Ex Malo Bonum' is translated as 'out of bad comes good'.

**Fig. 9:**
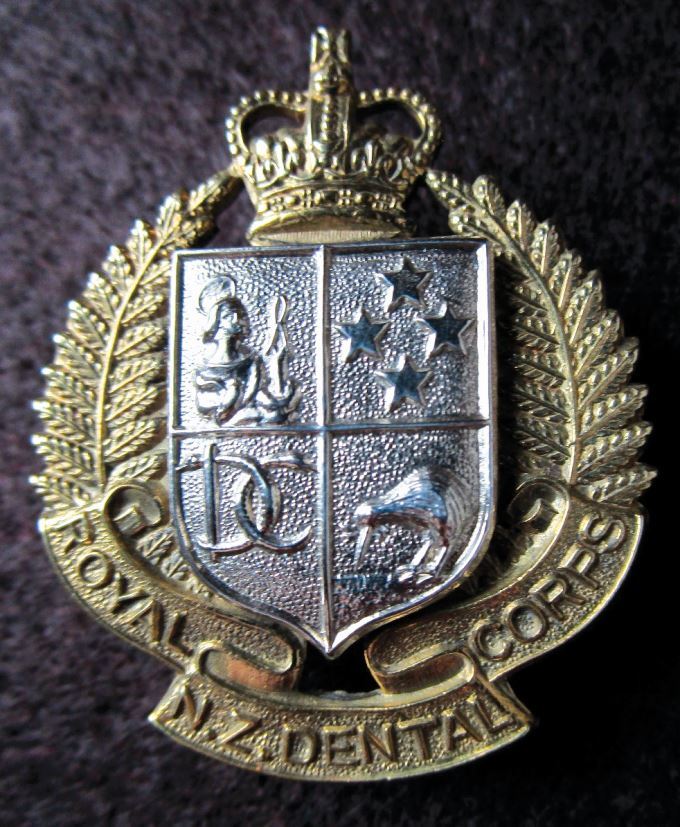
Cap badge of the Royal New Zealand Dental Corps in bimetal (1952, queen's crown). The shield in the centre of the badge has four elements: top left, an image of Saint Apollonius; top right, the stars of the Southern Cross constellation; bottom left, the original insignia of the Corps from 1915; and bottom right, the national bird of New Zealand, the kiwi. Note that the encircling wreath changed to the New Zealand fern rather than the laurel leaf wreath, common on British Army insignia and the insignia on the original badge (see Figure 8)

In 1947, the insignia changed to a much bolder design with imposing cap and collar badges. The design shows the ingenuity of the designer, combining the previous design with national and dental symbols (Fig. 9).

### Indian Army Dental Corps

**Fig. 10:**
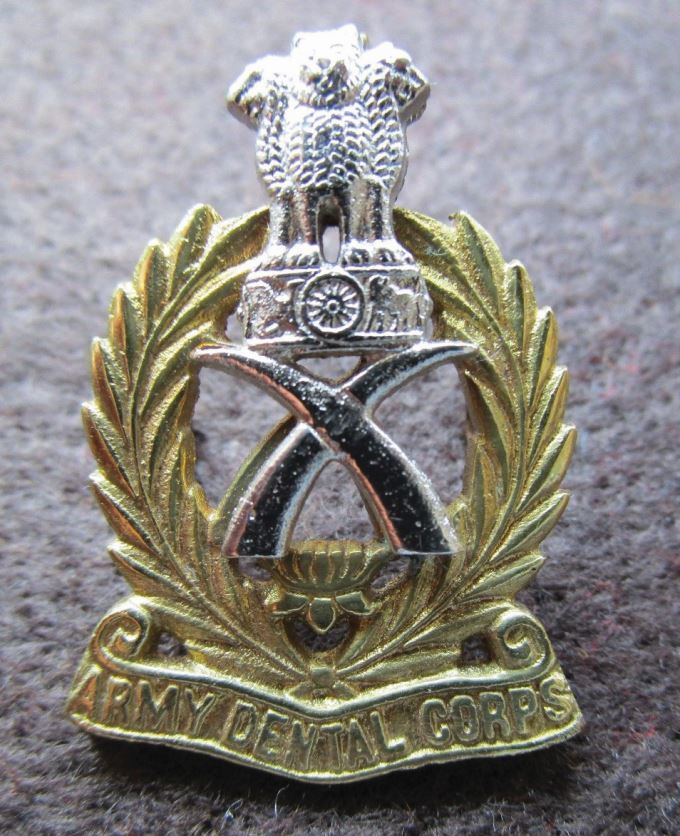
A cast Indian Army cap badge post-1950. The insignia above the elephant tusks is the 'Lion Capital', originally from the time of Emperor Ashoka the Great who ruled India (250 BCE), who is credited with the first written legislation. Ashoka was the first emperor who expanded his kingdom across India and erected pillars called the Ashoka stambh or Ashoka pillars. On top of this pillar sat four lions facing four directions. It was adopted as the official emblem of India in 1950^9^

Like the history of the Canadian Dental Corps, that of the Indian Dental Corps is complex.^7^ Briefly, initially dental care for Indian officers was provided by the British AD Corps. In 1941, a few civilian dentists were given emergency commissions and the Dental Branch of the Indian Medical Service was established. In 1943, the Indian Army Dental Corps was established, but in 1945 was merged back into the Indian Medical Corps. Subsequent to independence in 1947, the Corps was re-established as the Army Dental Corps with the current cap badge adopted in 1950 (Fig. 10).

## Conclusion

This paper provides some insights into the fascinating insignia of the British and Commonwealth Army Dental Corps. The RADC has a rich history and interrelationship with its equivalent corps in the Commonwealth. Insignia worn over the last 100 years, of the different countries' dental personnel, cleverly combines national and dental devices and provides attractive cap, collar and sweetheart badges. These were for members of the corps and for family left behind by military posting, but now also for collectors.
